# Deep learning reconstruction enhances 1.5T MR angiography beyond 3T in vascular visualization for Moyamoya disease

**DOI:** 10.1007/s11604-025-01945-9

**Published:** 2026-01-24

**Authors:** Ayako Omori, Hiroyuki Tatekawa, Tatsushi Oura, Natsuko Atsukawa, Shu Matsushita, Daisuke Horiuchi, Hirotaka Takita, Yasuhito Mitsuyama, Taro Shimono, Tsutomu Ichinose, Yusuke Watanabe, Takeo Goto, Yukio Miki, Daiju Ueda

**Affiliations:** 1 Department of Diagnostic and Interventional Radiology, Graduate School of Medicine, Osaka Metropolitan University, 1-4-3, Asahi-machi, Abeno-ku, Osaka, 545-8585 Japan; 2 Department of Neurosurgery, Graduate School of Medicine, Osaka Metropolitan University, Osaka, Japan; 3 Department of Artificial Intelligence, Graduate School of Medicine, Osaka Metropolitan University, Osaka, Japan

**Keywords:** Deep learning reconstruction, MRA, Moyamoya disease, Artificial intelligence

## Abstract

**Purpose:**

Deep learning reconstruction (DLR) is increasingly being applied to clinical magnetic resonance angiography (MRA); however, its evaluation across different magnetic field strengths in moyamoya disease is limited. This study assessed whether DLR affects visualization of stenotic arteries and collateral moyamoya vessels (MMVs).

**Materials and methods:**

Thirty-two patients (mean age, 40 years; male/female, 9/23) with suspected or confirmed moyamoya disease between 2015 and 2024 were retrospectively selected. All patients underwent time-of-flight MRA using 1.5T and 3T scanners within a 400-day interval. Differences in image quality and vascular visualization were assessed across four types of maximum intensity projection images from 1.5T and 3T MRA with and without DLR. The rankings of imaging quality and vascular depiction, Houkin classification scores, and visualization scores of the MMVs were compared using the Wilcoxon signed-rank test.

**Results:**

When all four groups (1.5T and 3T MRA with/without DLR of 32 patients) were simultaneously compared using ranking scores, both overall image quality and visualization of MMVs were consistently rated higher for the DLR-enhanced images; notably, the 1.5T DLR-enhanced MRA achieved higher quality rankings than the 3T original MRA (*p* < 0.048). Houkin’s scores significantly decreased in DLR-enhanced MRA (*p* < 0.016) when compared at the same field strength, indicating less severe stenosis in DLR-enhanced images. MMVs visualization scores tended to shift toward higher grades after DLR, although this difference was not significant.

**Conclusion:**

DLR significantly improved vascular visualization in moyamoya disease, with 1.5T DLR-enhanced MRA outperforming the original 3T MRA in terms of image quality and MMVs depiction.

**Supplementary Information:**

The online version contains supplementary material available at 10.1007/s11604-025-01945-9.

## Introduction

Moyamoya disease is a chronic cerebrovascular disorder characterized by progressive stenosis of the terminal portion of the internal carotid artery and development of abnormal collateral vessels [1, 2]. Magnetic resonance angiography (MRA) is commonly used to evaluate the degree of arterial stenosis and the visibility of peripheral vasculature and moyamoya vessels (MMVs) [3–6]. Previous studies investigating image reconstruction techniques, including parallel imaging and compressed sensing, have demonstrated their potential to accelerate MRA acquisition [7–10]. However, these methods often result in reduced image quality compared to conventional sequences. Recently, deep learning reconstruction (DLR) techniques have advanced remarkably and are increasingly being applied to magnetic resonance imaging (MRI), including MRA, offering potential improvements in image quality [11–13]. When integrated with conventional acceleration methods, DLR can improve image quality and reduce scan time, making high-resolution MRA more feasible in clinical practice.

Some studies have evaluated the effect of DLR on MRA and demonstrated its efficacy in enhancing image quality by reducing noise and improving vessel delineation [14, 15]. Although MRA with and without DLR have been compared, the effect of magnetic field strength on DLR-enhanced MRA remains unclear because DLR is typically applied during acquisition, limiting retrospective analysis. Recently, SwiftMR, a commercially available digital imaging and communications in medicine (DICOM)-based tool, has enabled the retrospective application of DLR by denoising and enhancing image resolution. This allows for a direct comparison of MRA quality across different field strengths using existing MRI data, supporting a more thorough evaluation of DLR effects [12]. However, the evaluation of DLR in different MRA settings remains limited, and no studies have applied DLR techniques to MRA, particularly in moyamoya disease, to assess their ability to improve the visualization of stenotic arteries and collateral MMVs.

Therefore, this study compared four sets of MRA, acquired at both 1.5T and 3T, with and without DLR, within approximately one year in patients with moyamoya disease. Specifically, we evaluated whether DLR-enhanced MRA acquired at 1.5T can achieve image quality comparable to or better than that of the original MRA acquired at 3T by assessing changes in vascular visualization using SwiftMR. The 3T MRI scanners are generally associated with superior vascular depiction on MRA compared with 1.5T scanners [6]. However, once this technique is established, it will be particularly beneficial for patients with physical limitations preventing 3T MRI or for institutions equipped only with 1.5T scanners, potentially enabling high-quality cerebrovascular assessment regardless of MRI system availability.

## Materials and methods

This study was approved by the ethics committee of our institution (IRB: 2025-022), and the requirement for informed consent was waived because of its retrospective design. This study complied with the principles of the Declaration of Helsinki and was structured according to the Strengthening the Reporting of Observational Studies in Epidemiology guidelines [16].

### Participants

**Fig. 1:**
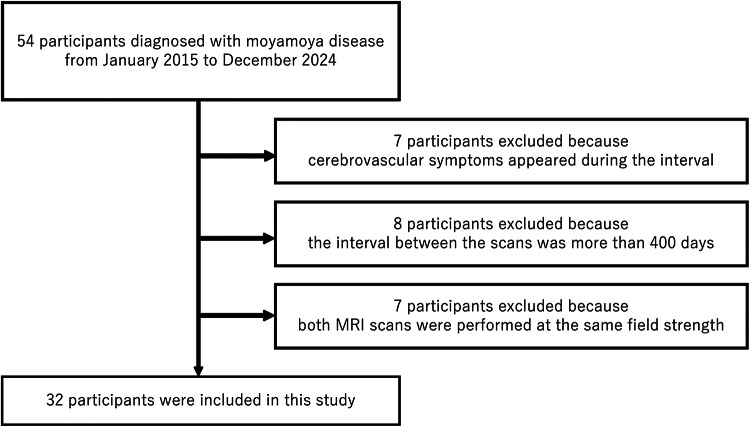
Selection flow chart

All MRA reports from January 2015 to December 2024 were retrospectively reviewed to identify participants diagnosed with or suspected of having moyamoya disease [17].Time-of-flight (TOF) MRA reports from participants who underwent both 1.5T and 3T MRI scans within an interval of 400 days were selected, regardless of which scan was performed first. This selection criterion was based on the fact that patients with asymptomatic moyamoya disease are typically followed up with MRA once a year at our institution. Participants were excluded if cerebrovascular symptoms appeared during the interval. Charts were also reviewed to ensure that no surgery occurred between the two MRI scans, and no postoperative MRI within three months were included. Finally, the earliest available pre- or post-operative data were extracted for each participant. The participant selection flowchart is shown in Fig. 1.

### MRI scanners and acquired data

MRI data were acquired using the following systems: 3T MRI scanners, including Achieva and Ingenia (Philips, Amsterdam, Netherlands) and Magnetom Vida (Siemens Healthineers, Erlangen, Germany), and 1.5T MRI scanners, including Achieva (Philips, Amsterdam, Netherlands) and Avanto (Siemens Healthineers, Erlangen, Germany). All scans followed clinically available standardized TOF MRA protocols to ensure consistency in data acquisition. None of the original TOF-MRA datasets were reconstructed using any vendor-native AI-based reconstruction tools. The detailed imaging parameters are summarized in Table 1.

**Table 1:** Imaging parameters of MR angiography protocol in five different scanners

Imaging parameters	Avanto	Achieva	Achieva	Ingenia	Magnetom Vida
Magnetic field strength	1.5T	1.5T	3T	3T	3T
Flip angle (degrees)	20	18	25	25	20
Echo time (ms)	7.15	6.91	3.45–5.76	3.45–5.76	3.69
Repetition time (ms)	24	19–23	23, 25	23, 25	21
Field of view (mm)	220 × 189 220 × 202 220 × 220	220 × 220	220 × 220	220 × 220	220 × 220
Slice thickness (mm)	0.7–0.75	1.1–1.3	1.1–1.5	1.1–1.25	0.5
Number of slices	143–208	160–275	150–250	170–260	262–300
Acquisition matrix	320 × 162	336 × 234–237	336 × 232 352 × 247	336 × 232	384 × 380
Reconstruction matrix	320 × 320	512 × 512	512 × 512	512 × 512	575 × 576
Reconstruction method	GRAPPA	SENSE	SENSE	SENSE	Compressed sensing
Acquisition time (s)	300–360	265–412	238–388	275–452	294–322
Number of participants	11	21	17	11	4

GRAPPA, Generalized auto calibrating partially parallel acquisitions; SENSE, Sensitivity encoding

### Deep learning reconstruction

TOF MRA data were acquired in the DICOM format, and a commercially available deep neural network-based MRI reconstruction software, SwiftMR (v3.0.11.1, AIRS Medical, Seoul, Korea), was applied retrospectively for image post-processing, enabling denoising and spatial resolution enhancement within the DICOM domain [12, 18]. This software supports both 2D and 3D acquisition across various anatomical regions, pulse sequences, contrast weightings, field strengths, and coil configurations. The noise-reduction level of 2 was applied in this study [19]. The resolution of all the post-DLR images was 768 × 768 pixels.

### Creation of maximum intensity projection images

Maximum intensity projection (MIP) images were generated from four time-of-flight MRA datasets—1.5T original, 1.5T DLR-enhanced, 3T original, and 3T DLR-enhanced—using Python (3.9.6) with SimpleITK, NumPy, and Pillow. For each dataset, volumes were rotated in 10° increments from − 90° to 90° about the head–foot and left–right axes to obtain coronal views, and about the anterior–posterior axis to obtain axial views. No editing was performed to remove the external carotid artery branches, to avoid bias in assessing the entire vasculature. Cross-acquisition intensities were harmonized by 3D histogram matching to the 3T DLR-enhanced reference. Volumes were converted to 32-bit floating points and processed with 128 histogram levels, seven quantile match points, and enabled mean-intensity thresholding. The harmonized volumes were then spatially standardized, and MIPs were rendered for reader assessment, yielding comparable background and vessel contrast across the four acquisitions.

### MRA evaluation

A comparative analysis between 1.5 T and 3T MRA scans with and without DLR was performed to assess the differences in image quality and vascular visualization. Three independent radiologists (A.O., T.O., and N.A., with 4, 6, and 10 years of experience in radiology, respectively) evaluated the MIP images while blinded to both the magnetic field strength and DLR status.

**Fig. 2:**
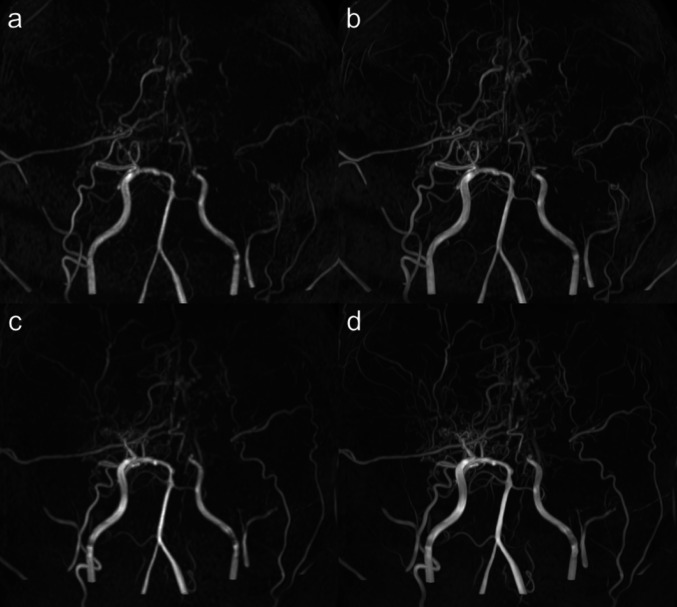
Representative case of a man in his 40s with moyamoya disease. The figure presents a 2 × 2 panel of zoomed maximum-intensity projection images: **a** 1.5T original, **b** 1.5T DLR-enhanced, **c** 3T original, and **d** 3T DLR-enhanced MRA. DLR clearly improves the visualization of moyamoya vessels. DLR, deep learning reconstruction; MRA, magnetic resonance angiography

Primary endpoint: for a within-subject ranking task, four composite MIP images per participant (1.5T original, 1.5T DLR-enhanced, 3T original, and 3T DLR-enhanced) were displayed simultaneously in a 2 × 2 panel. Panel positions were randomized for each case to conceal image identity, and readers were blinded to the field strength and DLR status. The radiologists ranked them according to overall image quality and vascular visualization, including depiction of major cerebral arteries and MMVs. A distinct ranking from the first to fourth was required for each case. Cases in which MMVs were not visible in any of the four images were excluded from MMV ranking analysis. An example of the four data comparisons is shown in Fig. 2, and additional examples from other cases are provided in the Supplemental Materials.

Secondary endpoint: for the scoring task, the three rotated MIP images of each dataset were merged into a single composite MIP, and the composite MIPs from all participants across the four acquisition types (1.5T original, 1.5T DLR–enhanced, 3T original, and 3T DLR-enhanced) were pooled and randomly ordered. Each image was graded according to Houkin’s classification of moyamoya disease staging [5]. Briefly, Houkin’s classification grades the internal carotid and middle cerebral artery into four categories: 0, normal; 1, stenosis of C1 (M1); 2, discontinuity of C1 (M1); and 3, invisible. The anterior and posterior cerebral arteries were graded into three categories: 0, normal A2 (P2) and distal branches; 1, signal decrease or loss in A2 (P2) and its distal branches; and 2, invisible. The total score of the four major cerebral arteries was used to determine the MRA grade: grade 1 (0–1), grade 2 (2–4), grade 3 (5–7), and grade 4 (8–10) for each hemisphere, with higher scores indicating more severe stenosis or arterial obstruction. The visibility of MMVs was also assessed using a 3-point scale: 0, few or no MMVs; 1, moderate number of MMVs (weak but sufficient visualization for diagnosis); and 2, large number of MMVs (clearly visualized) [8].

### Statistical analysis

As the four acquisition types (1.5T original, 1.5T DLR, 3T original, and 3T DLR) were categorically evaluated within subjects, within-subject nonparametric procedures were used. For the ranking task, the overall differences among the four acquisition types were analyzed using Friedman tests, followed by Wilcoxon signed-rank tests for the six pairwise comparisons with Holm correction. For ordinal scores (total score of Houkin’s classification and MMVs visibility), the right and left hemisphere values were averaged for each dataset before applying the same statistical procedures. For Wilcoxon signed-rank tests, the effect size was summarized as the rank-biserial correlation r_rb_. The inter-rater agreement for the ranking task across all four acquisition types was assessed using Kendall’s coefficient of concordance (W) with a 95% confidence interval (CI) from a nonparametric subject-level bootstrap (5000 resamples). Inter-rater reliability for the ordinal scores was evaluated using quadratic-weighted κ statistics. Because the scores were averaged across sides and expressed in 0.5-point increments, all values were multiplied by two to convert them into integer categories before calculating the coefficients. Because the three radiologists performed independent rankings and scoring and perfect concordance could not be assumed in advance, the results from one representative reader are presented in the main text, with the other two readers’ results provided in the Supplemental Materials. No formal sample size calculation or power analysis was performed; the sample size was determined based on the number of eligible patients available during the study period. Two-sided p values < 0.05 after multiplicity correction were considered statistically significant. Statistical analyses were conducted using GraphPad Prism version 10.4.1 software (GraphPad Software, San Diego, CA, USA).

## Results

Among the 54 participants diagnosed with or suspected of having moyamoya disease, 32 participants (mean age ± standard deviation, 40 ± 15.8 years; range, 8–70 years; males/females, 9/23) matched the eligibility criteria, and 128 sets of MIP images were evaluated by three radiologists. MRI scan with 1.5T was initially performed on 26 participants. Ten participants were in postoperative condition.

**Fig. 3:**
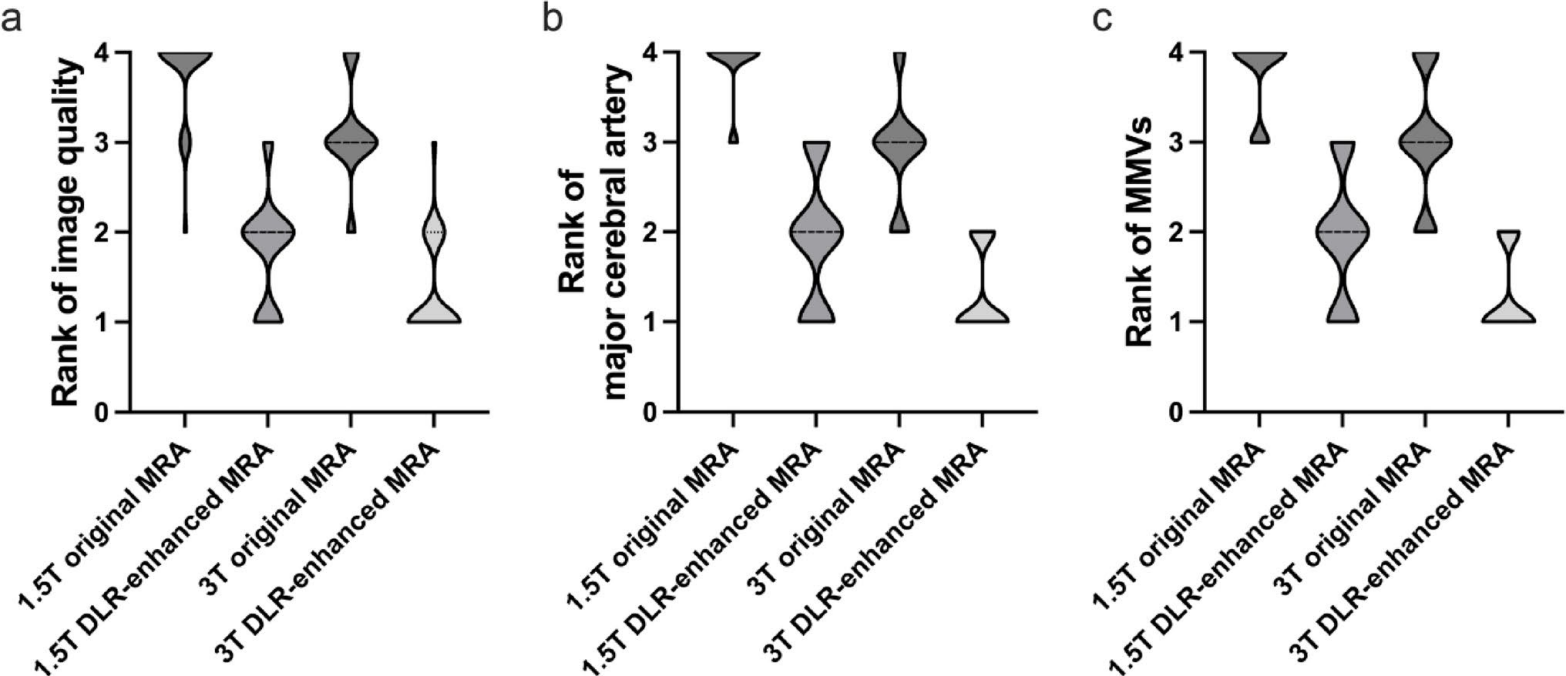
Violin plots showing the rank distributions for **a** overall image quality, **b** major cerebral artery visualization, and **c** moyamoya vessel visualization across the four acquisition types (1.5T original, 1.5T DLR-enhanced, 3T original, and 3T DLR-enhanced MRA). For reader 1, all pairwise comparisons of three analyses were statistically significant, with 3T DLR-enhanced MRA ranking highest, followed by 1.5T DLR-enhanced, 3T original, and 1.5T original MRA in descending order. DLR, deep learning reconstruction; MRA, magnetic resonance angiography

Across the three readers, the mean ranks for the 1.5T original, 1.5T DLR-enhanced, 3T original, and 3T DLR-enhanced MRA were 3.70, 1.91, 3.07, and 1.32 for overall image quality, respectively; 3.69, 2.00, 2.90, and 1.38 for the depiction of major cerebral arteries, respectively; and 3.57, 1.96, 2.90, and 1.31 for depiction of MMVs, respectively. Four of the 32 participants lacked MMVs in all four images and were excluded from the MMV ranking. For all ranking evaluations by the three readers, the Friedman test showed statistical significance (all *p* < 0.001). Across all readers, both the overall image quality and visualization of MMVs were consistently rated higher for the DLR-enhanced MRA than for the original MRA, even when comparing the 1.5T DLR-enhanced MRA with the 3T original MRA (*p* < 0.048; r_rb_ > 0.38). The depiction of major cerebral arteries was also consistently rated higher for DLR-enhanced MRA than for the original MRA by all three readers, although one reader showed no significant difference between the 1.5T DLR-enhanced MRA and 3T original MRA. The rankings of image quality and vascular depiction for reader 1 are shown in Fig. 3, and those for the other readers, raw ranking data, and pairwise effect sizes and p values are provided in the Supplemental Materials.

**Fig. 4:**
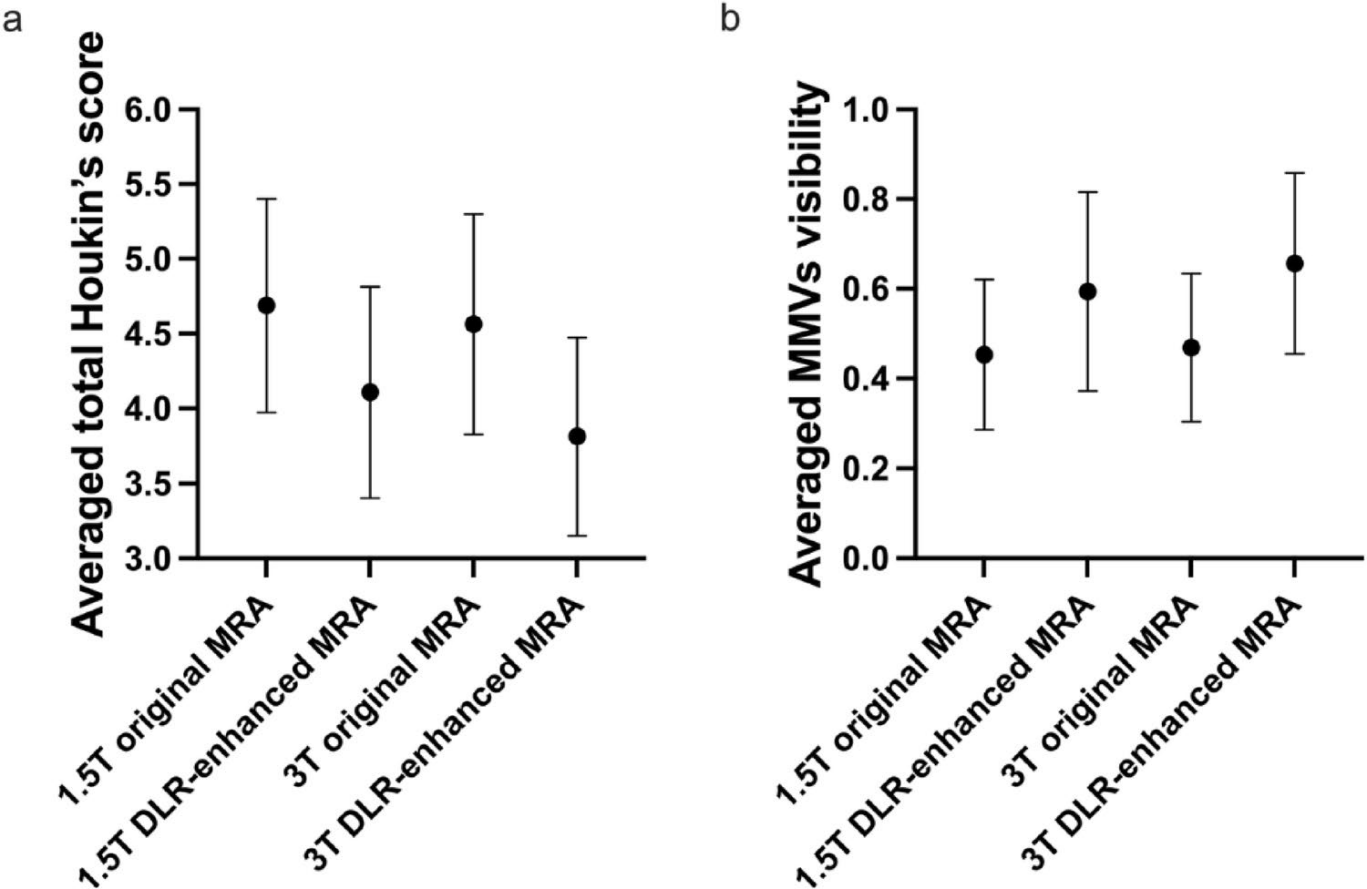
Mean scores with 95% confidence intervals for **a** total Houkin’s score and **b** MMVs visualization score. For reader 1, Houkin’s scores are significantly lower for DLR-enhanced MRA than for non-DLR-enhanced MRA at the same field strength. MMV visualization scores tend to be higher after DLR, although the differences do not reach statistical significance after Holm correction. DLR, deep learning reconstruction; MRA, magnetic resonance angiography; MMVs, moyamoya vessels

For the ordinal scoring evaluation, the mean values for the 1.5T original, 1.5T DLR-enhanced, 3T original, and 3T DLR-enhanced MRA across the three readers were 4.65, 4.06, 4.44, and 3.85 for the total Houkin’s classification score, respectively; and 0.22, 0.37, 0.31, and 0.50 for the MMVs visualization score, respectively. For the Friedman test, the Houkin’s classification was statistically significant for all readers (all *p* < 0.001), whereas the MMVs scoring evaluation did not show statistical significance for one of the three readers. Houkin’s scores significantly decreased in DLR-enhanced MRA compared with the original MRA (*p* < 0.005; r_rb_ < -0.62) at the same magnetic field strength. Visualization of MMVs tended to shift toward higher grades after DLR; however, for two readers, MMV scores were significantly higher for 3T DLR-enhanced MRA than for 1.5T original MRA, and no other significant differences between DLR-enhanced and original MRA were observed. Houkin’s classification and MMVs depiction for reader 1 are shown in Fig. 4, and those for the other readers, raw scoring data, pairwise effect sizes, and p values are provided in the Supplemental Materials.

Kendall’s W for the ranking task indicated substantial overall agreement across all four acquisition types with values of 0.70 (95% CI 0.63–0.78) for image quality, 0.61 (95% CI 0.52–0.72) for major cerebral artery visualization, and 0.60 (95% CI 0.51–0.71) for MMV visibility. The mean quadratic-weighted κ coefficient among the three readers for the scoring task was 0.77 for the total Houkin’s classification score and 0.32 for MMV visibility score. Pairwise κ for three readers are provided in the Supplemental Materials.

## Discussion

This study evaluated whether the DLR technique can improve the depiction of vessels on MRA by assessing the vascular visualization in moyamoya disease. When all four groups of 1.5T and 3T with/without DLR were simultaneously compared, both overall image quality and visualization of MMVs showed consistently higher rates for the DLR-enhanced images than those of the non-DLR images; notably, the 1.5T DLR-enhanced MRA achieved higher quality rankings than the 3T original MRA. When each group was evaluated separately, Houkin’s scores significantly decreased in DLR-enhanced MRA when compared at the same field strength. MMVs visualization tended to shift toward higher grades after DLR, although the difference was not statistically significant.

In this study, DLR-enhanced MRA was consistently ranked higher than non-DLR-enhanced MRA for overall image quality and visualization of MMVs, even across magnetic field strengths; notably, 1.5T DLR-enhanced MRA achieved higher quality rankings than 3T original MRA. A previous study using 1.5T has also shown noise reduction, improved vessel depiction, and sharper images with DLR, and another study found these benefits to be particularly pronounced at 1.5T compared with 3T images [12, 15]. In contrast to these earlier reports that did not assess the same patients across field strengths, the current study evaluated paired examinations of the same patients at both 1.5T and 3T, thereby minimizing inter-subject variability and enabling a direct comparison of DLR effects. Accordingly, the paired-subject design provides robust evidence that DLR can partially offset limitations associated with lower field strength, helping to bridge hardware-related differences in MRA image quality. Consistent with this concept, our results showed that even at 1.5T, DLR can outperform 3T non-DLR MRA for the visualization of moyamoya disease, which may be especially beneficial for patients unable to undergo 3T MRI or for institutions equipped only with 1.5T scanners.

Regarding the major cerebral arteries, Houkin’s score decreased with DLR compared with the original images. This classification evaluates the degree of stenosis or obstruction of major cerebral arteries. The observed reduction in scores should not be interpreted as disease regression but as an effect of improved image quality; signals were restored in vessels that previously appeared interrupted, allowing continuity visualization despite unchanged clinical status [12]. In contrast, MMV visibility scores tended to shift toward higher grades after DLR, and for two readers, MMVs scores were significantly higher for 3T DLR-enhanced MRA than for 1.5T original MRA. Previous computed tomography angiography studies have reported that DLR enables the reliable depiction of vascular continuity and may enhance the visualization of small vessels [20, 21]. Thus, it was confirmed that DLR’s ability to boost spatial resolution and suppress noise can also reveal small-caliber vessels, such as MMVs, that might be missed in conventional reconstructions for MRA.

However, the use of standardized imaging protocols was suggested for the evaluation of moyamoya disease, and caution is warranted regarding the differences in scanner equipment and magnetic field strength [6, 22]. The present findings further indicate that, in addition to scanner and field strength differences, the presence or absence of DLR also needs to be carefully considered when assessing vessel visualization.

This study has some limitations. First, the DLR-enhanced MRA was performed using a limited number of MRI scanners and participants from a single institution. Further investigations involving a wider variety of MRI systems and larger cohorts are warranted to validate and generalize these findings. Second, the MRI scanners used in this study were introduced at different time points. Although newer MRI scanners are generally associated with superior vascular depiction on MRA, all 3T scanners employed in this study were introduced after the 1.5T scanners, thereby minimizing this potential confounding factor. Third, cross-acquisition intensities were harmonized by histogram matching to a 3T DLR reference; a neutral reference or no-normalization arm was not implemented; thus, subtle bias toward DLR-like contrast cannot be excluded. Fourth, although no participant underwent surgery or experienced clinical deterioration between the two MRI examinations, the influence of subclinical vascular events cannot be entirely excluded and may have affected the results. Fifth, a reference standard such as digital subtraction angiography was not available in this study. Accordingly, the observed reduction in Houkin’s score after DLR reflects improved visualization on MRA but was not validated against angiographic findings.

In conclusion, DLR improved vascular visualization in moyamoya disease, with 1.5T DLR-enhanced MRA achieving superior image quality and MMVs depiction compared with 3T original MRA. This finding indicates that the DLR technique can provide high-quality cerebrovascular evaluation even at 1.5T, making it particularly valuable for patients who cannot undergo 3T MRI and for facilities equipped only with 1.5T scanners. However, evaluation of moyamoya disease requires careful consideration not only of scanner and field-strength differences, but also of whether DLR is used.

## Supplementary Information

Below is the link to the electronic supplementary material.

Supplementary Material 1

## Data Availability

Data cannot be shared openly but are available on request from authors.
