# Supplementary material for: Deep learning reconstruction enhances 1.5T MR angiography beyond 3T in vascular visualization for Moyamoya disease
Source: Jpn J Radiol. 2026 Jan 24;44(6):998–1005. doi: 10.1007/s11604-025-01945-9 (PMC13222166; doi:10.1007/s11604-025-01945-9)
Supplement: Supplementary file 1 — Supplementary Material 1 [file 11604_2025_1945_MOESM1_ESM.pdf]

Supplemental materials

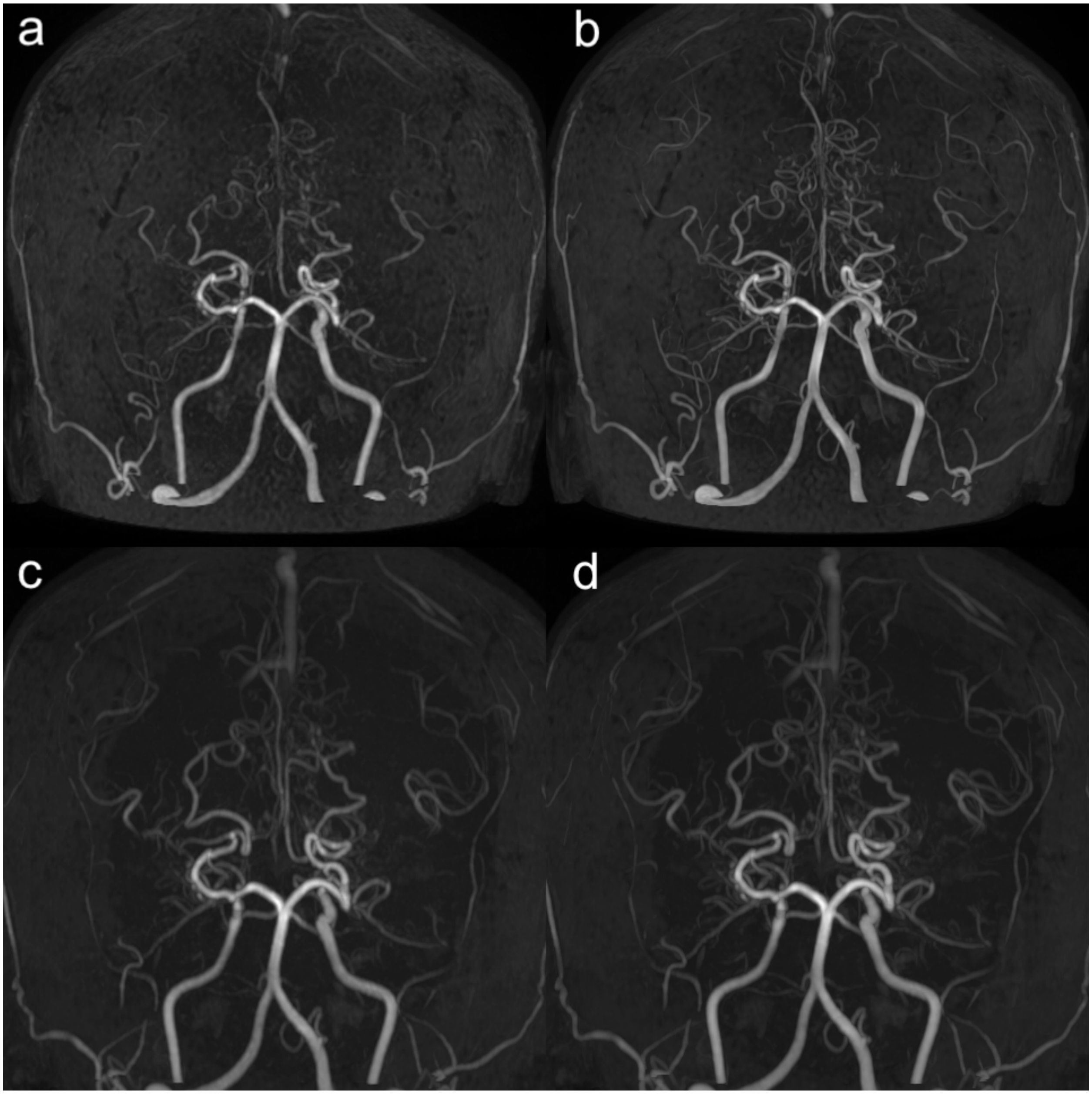

Supplemental Figure 1 Representative case of a woman in her 50s with moyamoya disease. The figure presents a 2×2 panel of zoomed maximum-intensity projection images: (a) 1.5T original, (b) 1.5T DLR-enhanced, (c) 3T original, and (d) 3T DLR-enhanced MRA. DLR clearly improves the visualization of moyamoya vessels. DLR, deep learning reconstruction; MRA, magnetic resonance angiography

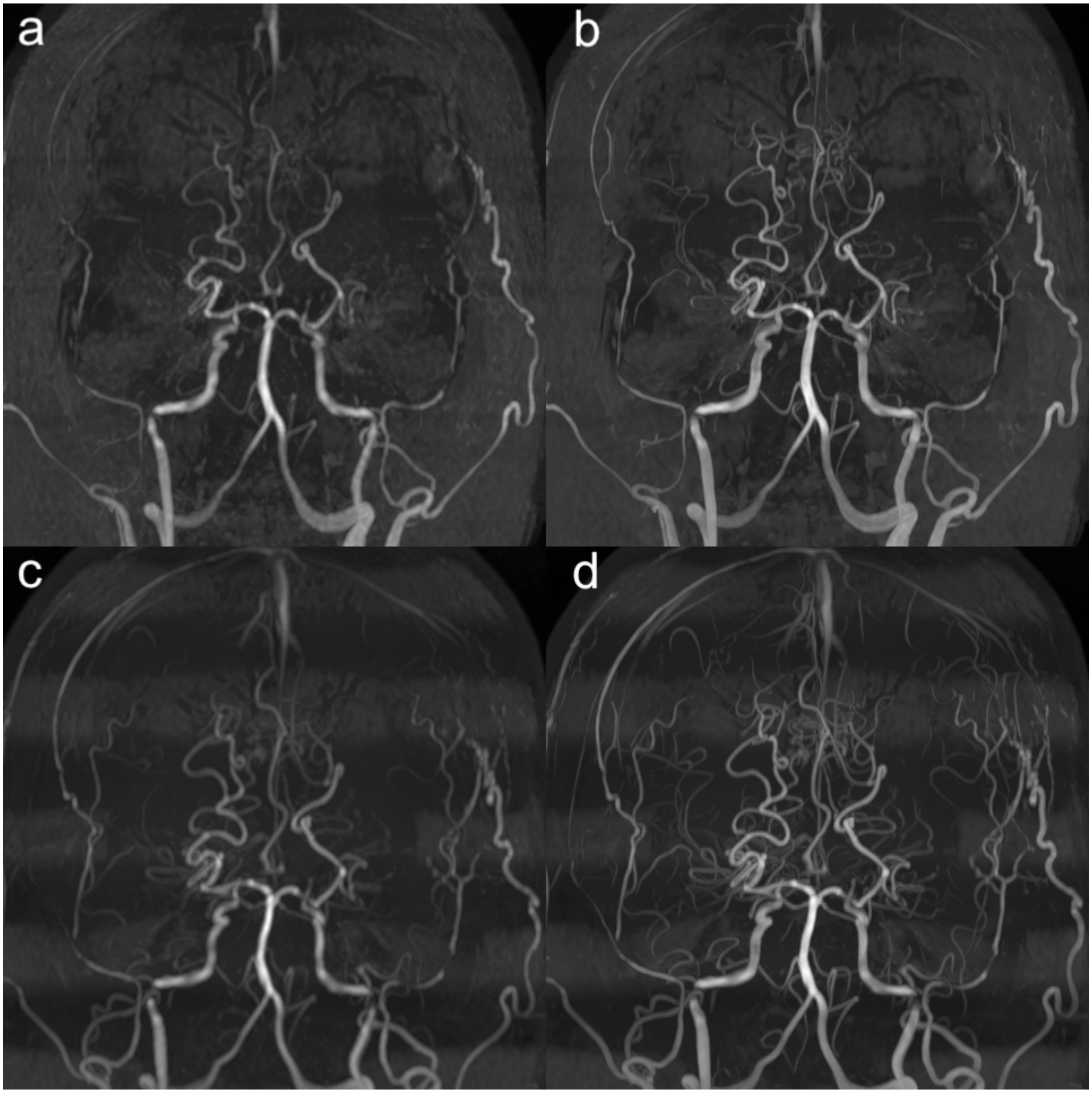

Supplemental Figure 2 Representative case of a woman in her 50s with moyamoya disease. The figure presents a 2×2 panel of zoomed maximum-intensity projection images: (a) 1.5T original, (b) 1.5T DLR-enhanced, (c) 3T original, and (d) 3T DLR-enhanced MRA. DLR clearly improves the visualization of moyamoya vessels. DLR, deep learning reconstruction; MRA, magnetic resonance angiography

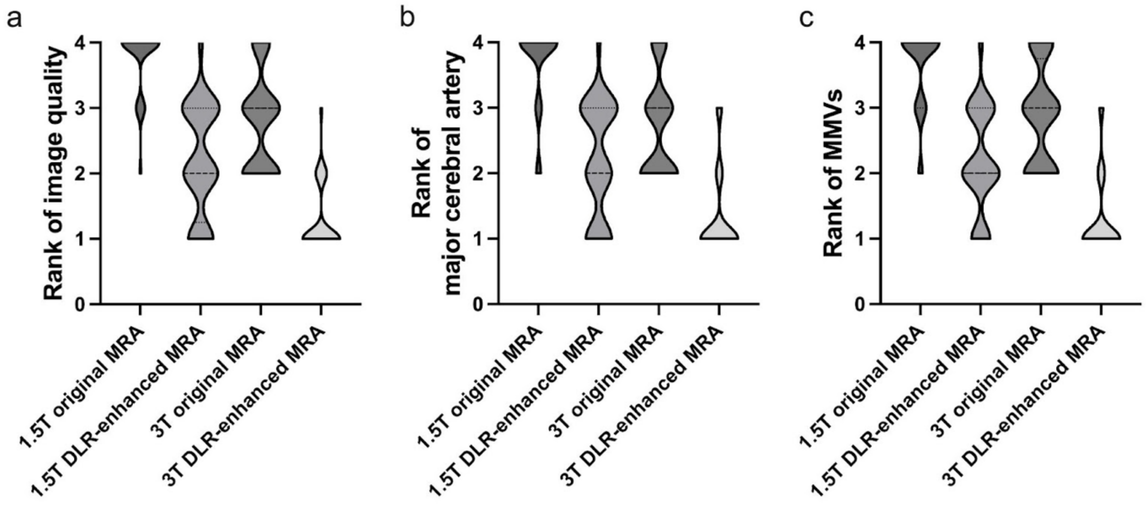

Supplemental Figure 3 Violin plots showing the rank distributions for (a) total image quality, (b) major cerebral artery visualization, and (c) MMVs visualization across the four acquisition types (1.5T original, 1.5T DLR-enhanced, 3T original, and 3T DLR-enhanced MRA) for reader 2. All DLR-enhanced images were consistently ranked higher than non-DLR-enhanced images, except for the evaluations of the major cerebral arteries between the 1.5T DLR-enhanced and 3T original images.

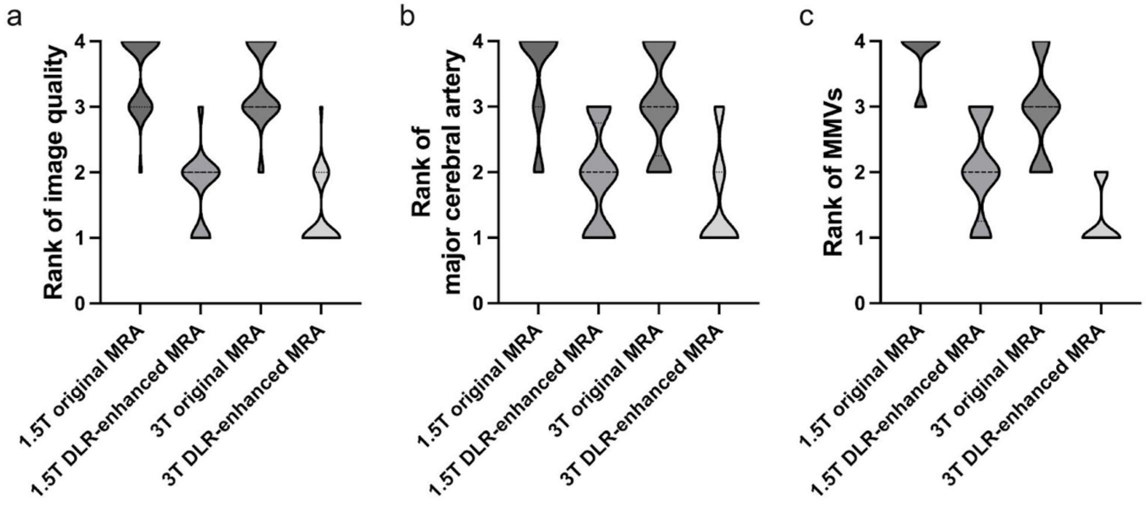

Supplemental Figure 4 Violin plots showing the rank distributions for (a) total image quality, (b) major cerebral artery visualization, and (c) MMVs visualization across the four acquisition types (1.5T original, 1.5T DLR-enhanced, 3T original, and 3T DLR-enhanced MRA) for reader 3. All DLR-enhanced images consistently ranked higher than non-DLR-enhanced images.

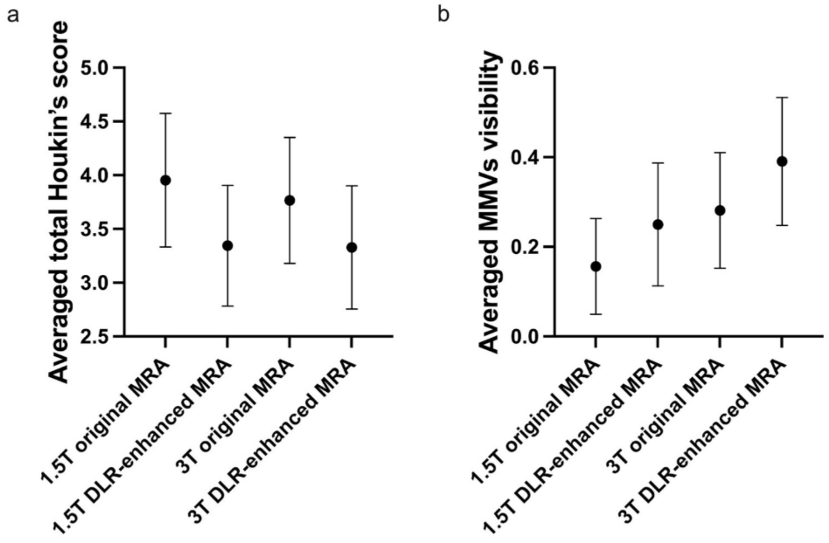

Supplemental Figure 5 Mean scores with 95% confidence intervals for (a) total Houkin's score and (b) MMVs visualization score for reader 2. Houkin's scores significantly decreased in the DLR-enhanced MRA compared to the original MRA at the same magnetic field strength. MMVs visibility scores were significantly higher for 3T DLR-enhanced MRA than for 1.5T original MRA, while no other significant differences were observed.

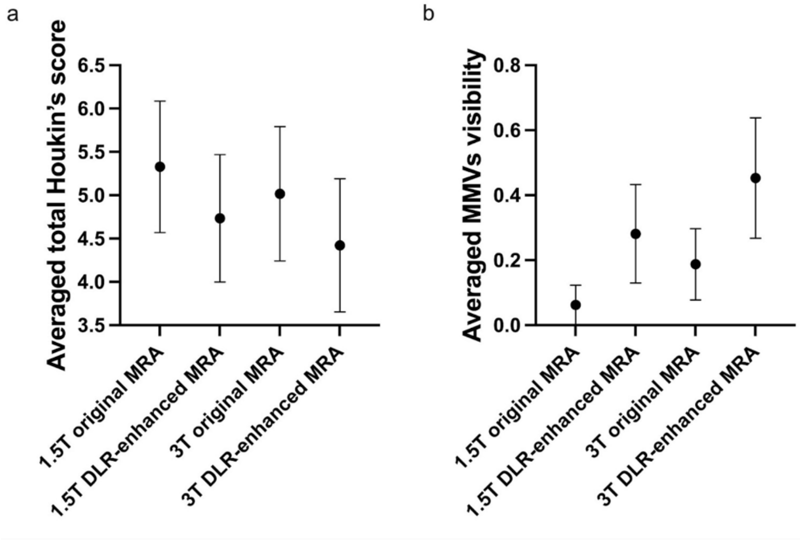

Supplemental Figure 6 Mean scores with 95% confidence intervals for (a) total Houkin's score and (b) MMVs visualization score for reader 3. Houkin's scores significantly decreased in the DLR-enhanced MRA compared with the original MRA at the same magnetic field strength. The visibility scores are significantly higher for DLR-enhanced MRA than for the original MRA, except when comparing 1.5T DLR-enhanced MRA with the original 3T MRA.

Supplemental Table 1 The ranks and scores of each reader for image quality, large cerebral artery, and MMVs, and Houkin's classification and MMVs visibility scoress

	1.5T original	1.5T DLR -enhanced	3T original	3T DLR -enhanced
**Reader 1**				
Image quality	3.78 (0.49)	1.78 (0.61)	3.09 (0.53)	1.34 (0.55)
Large cerebral artery	3.88 (0.34)	1.91 (0.73)	2.91 (0.59)	1.31 (0.47)
MMVs	3.75 (0.44)	1.93 (0.72)	3.04 (0.69)	1.29 (0.46)
Houkin's Total score	4.69 (1.98)	4.11 (1.95)	4.56 (2.04)	3.81 (1.84)
MMV visibility score	0.45 (0.46)	0.59 (0.61)	0.47 (0.46)	0.66 (0.56)
**Reader 2**				
Image quality	3.75 (0.51)	2.19 (0.86)	2.78 (0.75)	1.28 (0.52)
Large cerebral artery	3.69 (0.64)	2.19 (0.90)	2.72 (0.77)	1.31 (0.64)
MMVs	3.64 (0.62)	2.18 (0.82)	2.89 (0.79)	1.29 (0.60)
Houkin's total score	3.95 (1.72)	3.34 (1.56)	3.77 (1.62)	3.33 (1.59)
MMV visibility score	0.16 (0.30)	0.25 (0.38)	0.28 (0.36)	0.39 (0.39)
**Reader 3**				
Image quality	3.56 (0.56)	1.75 (0.57)	3.34 (0.60)	1.34 (0.55)
Large cerebral artery	3.50 (0.76)	1.91 (0.77)	3.09 (0.77)	1.50 (0.76)
MMVs	3.79 (0.42)	2.04 (0.74)	2.93 (0.72)	1.25 (0.44)
Houkin's total score	5.33 (2.11)	4.73 (2.04)	5.02 (2.15)	4.42 (2.13)
MMV visibility score	0.06 (0.17)	0.28 (0.42)	0.19 (0.30)	0.45 (0.51)

Data are presented as mean values, with standard deviations in parentheses.

Supplemental Table 2 Pairwise effect sizes (upper) and *p* values (lower) of rank of image quality, large cerebral artery, and MMVs, and Houkin's classification and MMVs visibility scores

	Reader 1			Reader 2			Reader 3		
	1.5T DLR -enhanced	3T original	3T DLR -enhanced	1.5T DLR -enhanced	3T original	3T DLR -enhanced	1.5T DLR -enhanced	3T original	3T DLR -enhanced
**Image quality**									
1.5T original	-1<br>< .001*	-0.61<br>.001*	-0.99<br>< .001*	-0.97<br>< .001*	-0.71<br>< .001*	-0.99<br>< .001*	-1<br>< .001*	-0.2<br>.32	-0.98<br>< .001*
1.5T DLR-enhanced		0.88<br>< .001*	-0.39<br>.045*		0.38<br>.048*	-0.65<br>< .001*		0.92<br>< .001*	-0.37<br>.057
3T original			-1<br>< .001*			-1<br>< .001*			-1<br>< .001*
**Large cerebral artery**									
1.5T original	-1<br>< .001*	-0.8<br>< .001*	-1<br>< .001*	-0.97<br>< .001*	-0.64<br>.001*	-0.96<br>< .001*	-1<br>< .001*	-0.28<br>.16	-0.89<br>< .001*
1.5T DLR-enhanced		0.7<br>< .001*	-0.51<br>.006*		0.31<br>.11	-0.6<br>.003*		0.67<br>< .001*	-0.28<br>< .001*
3T original			-1<br>< .001*			-1<br>< .001*			-1<br>< .001*
**MMVs**									
1.5T original	-1<br>< .001*	-0.6<br>.003*	-1<br>< .001*	-0.97<br>< .001*	-0.55<br>.008*	-0.97<br>< .001*	-1<br>< .001*	-0.69<br>< .001*	-1<br>< .001*
1.5T DLR-enhanced		0.72<br>< .001*	-0.55<br>.006*		0.43<br>.039*	-0.62<br>.003*		0.57<br>.005*	-0.64<br>.001*
3T original			-1<br>< .001*			-1<br>< .001*			-1<br>< .001*
**Houkin's total score**									
1.5T original	-0.75<br>.003*	-0.1<br>.67	-0.78<br>< .001*	-0.87<br>< .001*	-0.31<br>.18	-0.68<br>.002*	-0.64<br>.004*	-0.4<br>.08	-0.74<br>< .001*
1.5T DLR-enhanced		0.39<br>.09	-0.23<br>.31		0.6<br>.011*	-0.08<br>.73		0.34<br>.15	-0.34<br>.15
3T original			-0.62<br>.005*			-0.64<br>.004*			-0.69<br>.002*
**MMV visibility score**									
1.5T original	0.43<br>.13	0.01<br>.99	0.57<br>.048	0.57<br>.21	0.58<br>.08	0.7<br>.006*	0.88<br>.004*	1<br>.016*	1<br>< .001*
1.5T DLR-enhanced		-0.43<br>.11	0.16<br>.61		0.2<br>.78	0.5<br>.10		-0.5<br>.18	0.63<br>.029
3T original			0.5<br>.07			0.75<br>.10			1<br>< .001*

Effect size: each cell reports [column condition] − [row condition], $r_{rb} > 0$ favors the column condition and $r_{rb} < 0$ favors the row condition; * denotes statistical significance after Holm correction.

Supplemental Table 3 Pairwise quadratic-weighted κ coefficient among the three readers

	Reader 1 vs. Reader 2	Reader 1 vs. Reader 3	Reader 2 vs. Reader 3
Houkin's total score	0.77 (0.71–0.83)	0.83 (0.78–0.88)	0.70 (0.64–0.76)
MMVs visibility score	0.24 (0.10–0.39)	0.30 (0.15–0.46)	0.42 (0.24–0.56)

Data are presented with 95% confidence intervals in parentheses.
